# Using multi-parametric quantitative MRI to screen for cardiac involvement in patients with idiopathic inflammatory myopathy

**DOI:** 10.1038/s41598-022-13858-y

**Published:** 2022-06-14

**Authors:** Lu Huang, Qian Tao, Peijun Zhao, Suqiong Ji, Jiangang Jiang, Rob J. van der Geest, Liming Xia

**Affiliations:** 1 Department of Radiology, Tongji Hospital, Tongji Medical College, Huazhong University of Science and Technology, Jiefang Avenue 1095, Wuhan, 430030 People’s Republic of China; 2 Department of Radiology, Leiden University Medical Center, Leiden, The Netherlands; 3 Department of Neurology, Tongji Hospital, Tongji Medical College, Huazhong University of Science and Technology, Wuhan, 430030 People’s Republic of China; 4 Department of Cardiology, Tongji Hospital, Tongji Medical College, Huazhong University of Science and Technology, Wuhan, 430030 People’s Republic of China

**Keywords:** Cardiovascular diseases, Diagnostic markers, Musculoskeletal system, Rheumatic diseases

## Abstract

Idiopathic inflammatory myopathies (IIM) is a group of heterogeneous autoimmune systemic diseases, which not only involve skeletal muscle but also myocardium. Cardiac involvement in IIM, which eventually develops into heart failure, is difficult to identify by conventional examinations at early stage. The aim of this study was to investigate if multi-parametric cardiac magnetic resonance (CMR) imaging can screen for early cardiac involvement in IIM, compared with clinical score (Myositis Disease Activity Assessment Tool, MDAAT). Forty-nine patients of IIM, and 25 healthy control subjects with comparable age-range and sex-ratio were enrolled in this study. All subjects underwent CMR examination, and multi-slice short-axis and 4-chamber cine MRI were acquired to evaluate biventricular global circumferential strain (GCS) and global longitudinal strain (GLS). Native T1 and T2 mapping were performed, and post-contrast T1 mapping and LGE were acquired after administration of contrast. A CMR score was developed from native T1 mean and T2 mean for the identification of cardiac involvement in the IIM cohort. Using contingency tables MDAAT and CMR were compared and statistically analyzed using McNemar test. McNemar’s test revealed no significant difference between CMR score and MDAAT (*p* = 0.454). CMR score had potential to screen for early cardiac involvement in IIM patients, compared to MDAAT.

## Introduction

Idiopathic inflammatory myopathies (IIM) is a group of heterogeneous autoimmune systemic diseases characterized by skeletal muscle inflammation, including polymyositis (PM), dermatomyositis (DM), immune-mediated necrotizing myopathy (IMNM) and inclusion body myositis (IBM)^1,2^. Cardiac involvement in IIM, occurring in around 75% of IIM patients, is an important extramuscular manifestation and a major cause of mortality in PM and DM as it eventually develops into heart failure^3,4^. However, about 70% of patients only have subclinical involvement^5,6^, which fails to be identified at an early stage due to lack of sensitive screening tools. Early detection of cardiac involvement in IIM is essential for timely treatment to prevent further adverse remodelling and cardiac mortality in this patient group.

Endomyocardial biopsy is considered as the golden standard to diagnose cardiac involvement in IIM, but it is limited in clinical practice due to its invasiveness and risk of complication. In clinical practice, cardiac involvement in IIM is usually diagnosed by the Myositis Disease Activity Assessment Tool (MDAAT)^7–10^. This method needs various time consuming and costly clinical examinations, including cardiac-specific serological markers, electrocardiography and echocardiography, but lacks the sensitivity to screen for subclinical cardiac involvement in IIM. Once symptoms appear in IIM patients, most of them has morphological or functional changes, which progress rapidly and even lead to heart failure. To improve the sensitivity of cardiac involvement in IIM patients, advanced diagnostic tools are of significant clinical interest.

Cardiac magnetic resonance imaging (CMR) is an advanced tool for evaluation of cardiac morphology, function, and tissue characteristics^11^. Late gadolinium enhancement (LGE) can detect focal fibrosis in the myocardium and is a highly specific indicator of cardiac injury in IIM. However, it is also lacking in sensitivity, as IIM patients without focal enhancement may still have myocardial involvement in the form of diffuse fibrosis^4^, which is not detectable by LGE MRI. In previous studies, it was reported that LGE only presents regions of focal hyper-enhancement in 19–35% of patients with IIM^12,13^. In addition, LGE requires administration of Gadolinium-based contrast agents (GBCA), which limits its use in IIM patients with GBCA allergy and renal dysfunction.

With the recent MR T1 and T2 mapping techniques, diffuse fibrosis and edema can be quantitatively assessed^14^. Previous studies have shown that up to 90% of LGE-negative IIM patients actually had elevated T1 and T2 values compared with normal^14,15^, which can reflect disease progression. Furthermore, it has also been reported that myocardial strain assessed by CMR or echocardiography can reveal subtle myocardial remodelling in different patient cohorts with preserved left ventricular ejection fraction (LVEF)^16,17^, thereby more sensitive than traditional imaging markers for detecting subclinical myocardial involvement.

We hypothesize that multiple relevant CMR parameters, including the tissue and mechanical properties of the myocardium as quantified by T1, T2, and strain, may improve the screening performance of cardiac involvement detection in IIM patients. The purpose of this study was therefore to develop a multi-parametric CMR score for the detection of subclinical myocardial involvement in IIM patients.

## Methods

### Study population

This study was approved by the local Institutional Review Board of Tongji Hospital, Tongji Medical College, Huazhong University of Science and Technology (TJ-C20121221), and written informed consent was obtained from all participating subjects. It complies with the ethical principles of the Declaration of Helsinki. A cohort of 57 consecutive patients with confirmed idiopathic inflammatory myopathy (IIM) screening for myocardial involvement were enrolled in this study from October 2015 to October 2020. All patients underwent skeletal muscle biopsy, laboratory tests of blood, muscle and myocardium enzyme, and myositis—specific as well as myositis-associate autoantibodies, electromyography, electrocardiography, echocardiography, and CMR. Some IIM patients were included in a previous study of our team published before^15^.

Patients with PM and DM were diagnosed according to the Bohdan and Peter criteria^18,19^ and the recommended diagnostics by the International Myositis Classification Criteria Project (IMCCP)^2^. Patients with immune-mediated necrotizing myopathy (IMNM) were diagnosed according to the ENMC criteria^8^. Cardiac involvement in IIM was diagnosed using the Myositis Disease Activity Assessment Tool (MDAAT)-cardiac domain^20^. Assessment of pericarditis, myocarditis, arrhythmia and sinus tachycardia were based on clinical manifestation, hs cTnI, electrocardiography, echocardiography, and conventional CMR. Pericarditis was diagnosed according to the 2015 ESC Guidelines for the diagnosis and management of pericardial diseases^21^, in patients who satisfied at least 2 of 4 diagnostic criteria. Patients who met clinical diagnostic criteria^22^ proposed by the European Society of Cardiology Working Group on Myocardial and Pericardial Diseases were defined as clinically suspected myocarditis. Arrhythmia (such as new-set conduction defects, atrial dysrhythmias, ventricular dysrhythmias and non-specific ST-T abnormalities) and sinus tachycardia (i.e. sinus rhythm with heart rate more than 100 beats per minute) were diagnosed based on electrocardiography.

**Figure 1:**
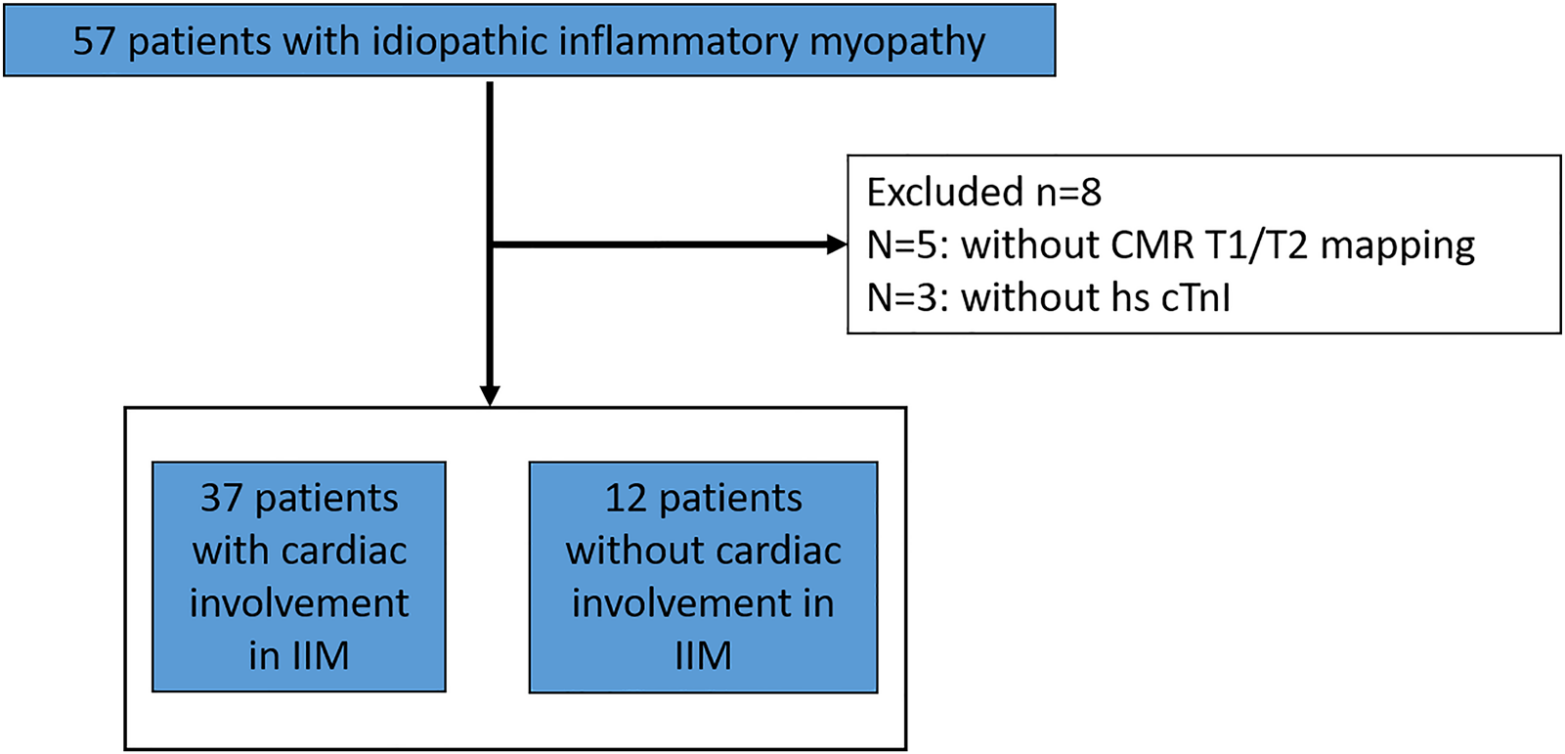
Flow chart of the enrolment procedure in this study.

Cardiac involvement was defined as cardiac visual analogue scales (VAS) on the MDAAT > 0, which was based on diagnosis and disease activitiy assessment of pericarditis, myocarditis, arrhythmia and sinus tachycardia^9^. Disease activity of cardiac involvement was evaluated by visual analogue scale (0–10 cm), with 0 representing no activity, 5 representing moderate activity, and 10 representing severe activity^23^. Exclusion criteria included: (1) history of coronary artery disease, myocarditis, cardiomyopathy, and valvular heart disease, (2) MR contraindication, (3) history of allergy to Gadolinium-based contrast agent, and (4) pregnancy. A flowchart of patient enrolment is shown in Fig. 1. Healthy subjects with similar age-range and sex-ratio were selected from a database of healthy subjects without cardiovascular disease, metabolic and systemic inflammation, were enrolled as the control group.

### CMR acquisition

All subjects underwent CMR imaging on a 3T MR scanner (Skyra, Siemens Healthineers, Erlangen, Germany) using an 18-channel cardiac coil. All images were acquired with retrospective ECG gating during breath-hold. The CMR protocol consisted of 4-chamber steady-state-free-precession (SSFP) cine and a stack of short-axis SSFP cine, short-axis native/post-contrast T1 mapping, T2 mapping and late gadolinium enhancement (LGE) imaging. Typical parameters of SSFP were: echo time (TE) = 1.39 ms, repetition time (TR) = 2.50 ms, field of view (FOV) = 320–360 mm, matrix = 192 × 146, flip angle (FA) = 47°, slice thickness = 8 mm, slice gap = 2 mm. The T1/T2 mapping and LGE sequences had the same imaging plane as the short-axis cine.

Native T1 mapping was acquired using an ECG-gated single-shot modified Look-Locker inversion recovery (MOLLI) sequence. The scan protocol was 5(3)3, with TE = 1.2 ms, TR = 322.48 ms, FOV = 320–360 mm, matrix = 192 × 144, FA = 35°, slice thickness = 8 mm, 72 segments, minimum TI = 100 ms, TI increment = 80 ms, GRAPPA acceleration factor = 2, imaging window = 136 ms. The T2 mapping technique involved a T2 prepared pulse sequence to produce single-shot T2 prepared SSFP images (T2p-SSFP), with different T2 preparation times (0 ms, 30 ms and 55 ms). The images were generated with three heart-beat recovery durations for each T2 preparation (total acquisition time of nine heartbeats). LGE imaging was performed 10–15 min after intravenous administration of gadobenate dimeglumine (0.2 mmol/kg, MultiHance, Bracco, Shanghai) using a phase-sensitive inversion-recovery (PSIR) sequence (TR = 777 ms, TE = 1.42 ms, matrix = 224 × 156, FA = 40°). Post-contrast T1 mapping was performed after 15 min following the administration of gadolinium using the MOLLI scan protocol of 4(1)3(1)2. All native T1, T2 mapping and post-contrast T1 mapping were acquired on the whole LV, which were same to short-axis SSFP cine. Haematocrit (HCT) was measured within 3 days of CMR scanning for extracellular volume (ECV) calculation.

### CMR data analysis

All CMR images were analysed by two experienced radiologists (L.H. and P.Z.) using the MASS research software (version 2019, Leiden University Medical Centre, Leiden, The Netherlands). LV/RV volumetric and functional parameters, including end-diastolic volume (EDV), end-systolic volume (ESV), stroke volume (SV), ejection fraction (EF), and mass were evaluated by manually drawing the endocardial and epicardial contours at the end-diastolic and end-systolic phases of the short-axis cine images. All volumes and masses were normalized by the body surface area (BSA).

**Figure 2:**
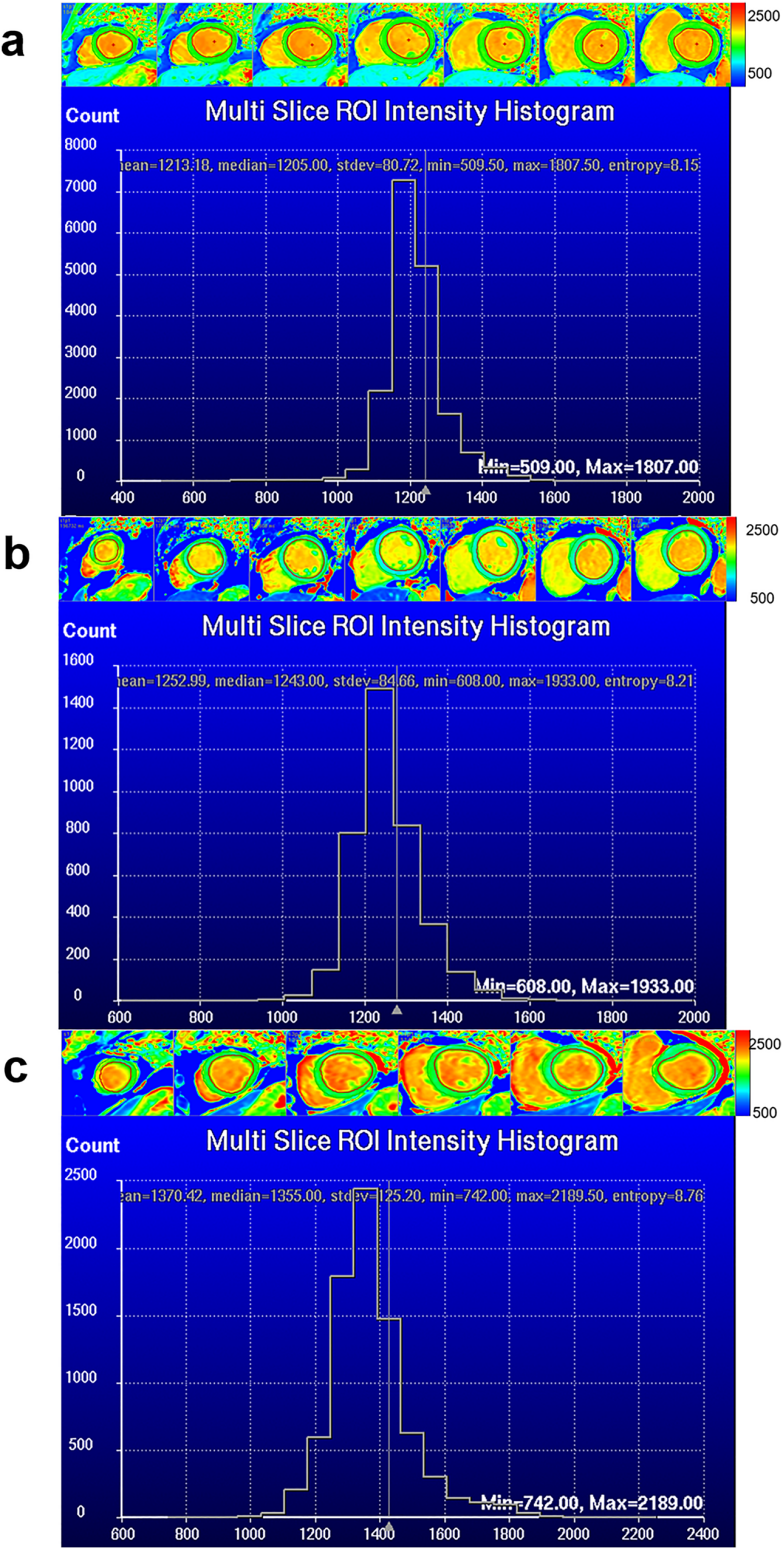
All slices of native T1mapping images and histograms of controls (**a**), IIM-C-(**b**) and IIM-C + (**c**) groups.

For both T1 and T2 mapping, a built-in algorithm was applied for motion correction by the MR scanner before parametric fitting. Examples of segmentation and histograms are shown in Fig. 2. Native T1 and post-contrast T1 of myocardium and blood pool were used to derive the ECV as follows:

$${\text{ECV}} = \, \left( {{1} - {\text{HCT}}} \right) \, \times \, \left( {{1}/\left( {{\text{post}}\,{\text{T1}}\,{\text{myocardium}}} \right) - {1}/\left( {{\text{native}}\,{\text{T1}}\,{\text{myocardium}}} \right)} \right)/\left( {{1}/\left( {{\text{post}}\,{\text{T1}}\,{\text{blood}}\,{\text{pool}}} \right) - {1}/\left( {{\text{native}}\,{\text{T1}}\,{\text{blood}}\,{\text{pool}}} \right)} \right)$$

Focal scar within the myocardium was categorized as present or absent on the LGE images by two observers in consensus after reading all PSIR images independently. A senior observer (L.X., with 21 years’ experience in CMR) adjudicated any discrepancies between two observers.

Automated deformable image registration was used to calculate LV/RV longitudinal strain from the manually traced endocardial contours at the end-diastolic phase of the 4-chamber cine images and biventricular circumferential strain was derived from the short-axis cine acquisition in all slices. Global strain values were averaged over all slices.

### Statistical analysis

Statistical analysis was performed using SPSS (version 23.0, Chicago, IL). Categorical variables were expressed as counts (percentage), and continuous variable as mean ± SD or median (interquartile range). Normality of distribution was tested using the Shapiro–Wilk test. We differentiated three categories of subjects: IIM patients with myocardial involvement (n = 37), IIM patients without myocardial involvement (n = 12), and healthy controls (n = 25). Comparisons among IIM patients with/without myocardial involvements and controls were performed by the analysis of variance (ANOVA) for normally distributed or Kruskal–Wallis test for non-normally distributed variables. Comparison between any of the two classes were performed by t-test (normal distribution) or Mann–Whitney U test (non-normal distribution) in case of continuous variables, or χ^2^ test for categorical variables. A CMR score was developed to screen for cardiac involvement in IIM, which was defined as pathological if T1 mean and/or T2 mean is above the 95% quantile in the healthy volunteers. If either of the patient's T1 and T2 values was above the 95% percentile, the score was defined as 1, and if both T1 and T2 value were above the 95% percentile the score was defined as 2, while neither of T1 and T2 value were above the 95% percentile the score was defined as 0. The CMR score > 1 was defined as "abnormal T1 or T2 value", i.e. cardiac involvement. The comparison between MDAAT and CMR score with their dichotomous answers were used to construct contingency tables and statistically evaluated using McNemar test. For McNemar’s test, binary ordinal cardiac involvement data was generated for each methods and transferred to a contingency table. Receiver operating characteristic (ROC) curve analysis was used to assess the screening sensitivity of T1/T2 mapping parameters to detect cardiac involvement in IIM. A P value lower than 0.05 was considered statistically significant.

## Results

### Subject characteristics

Forty-nine IIM patients were finally enrolled in this study, including 37 patients with myocardial involvement (denoted hereafter as IIM-C^+^) using the clinical criteria (MDAAT) as detailed in “Methods”, and 12 patients without myocardial involvement group (IIM-C^-^). Twenty-five healthy volunteers with similar age-range (42 ± 13 years, range 24–60 years, *p* = 0.886) and similar sex-ratio [female 16 (64%), *p* = 0.414], without cardiovascular disease, metabolic diseases and systemic inflammation, were enrolled as the control group.

Baseline characteristics of IIM patients, including diagnostic subgroups, serological markers and myocardial involvement assessment, are summarized in Table 1. In the group of IIM-C^+^ patients, 95% had no clinically significant myocardial symptoms (one with chest pain and one with palpitations). Heart rate of IIM-C^+^ was higher than controls [86 ± 19 bpm vs 73 ± 11 bpm; *p* = 0.026]. Blood hs cTnI in IIM-C^+^ patients’ group was higher than in IIM-C^-^ patients [median 5.9 pg/ml (IQR 1.9–25.9) vs median 2.2 pg/ml (IQR 1.9–4.3), *p* = 0.011]. Fourteen of 37 (38%) IIM-C^+^ patients had elevated hs cTnI level (upper limits is more than 15.4 pg/ml for female, and 34.2 pg/ml for male). The other serological biomarkers (e.g. NT–proBNP, CK, CK-MB, myoglobin, Lactate dehydrogenase, and hs CRP) showed no significant difference between IIM-C^+^ and IIM-C^-^ groups (*p* > 0.05, Table 1).

**Table 1:** Baseline characteristics of IIM patients.

	All IIM patients (n = 49)	IIM patients without Cardiac involvement (n = 12)	IIM patients with Cardiac involvement (n = 37)	*p* value
**Diagnostic subgroups**				–
PM, n (%)	30 (61)	7 (58)	23 (62)	
DM, n (%)	4 (8)	0 (0)	4 (11)	
IMNM, n (%)	14 (29)	4 (33)	10 (27)	
IBM	1 (2)	1 (9)	0 (0)	
Age, years	42 ± 16	37 ± 17	43 ± 16	0.712
Female, n (%)	37 (76)	9 (75)	28 (76)	0.962
BMI, kg/m^2^	22 ± 5	22 ± 3	22 ± 5	0.650
Heart rate, bpm	85 ± 17	82 ± 6	86 ± 19	0.843
Duration of IIM, median (IQR), years	0.4 (0.3–1.0)	0.8 (0.3–4.0)	0.4 (0.2–1.0)	0.065
hs cTnI, pg/ml	4.3 (1.9–21.7)	2.2 (1.9–4.3)	5.9 (1.9–25.9)	**0.011**
NT–proBNP, pg/ml	81.0 (36.0–208.8)	24.0 (23.0–39.0)	79.0 (34.8–261.0)	0.226
CK, U/L	1238.0 (231.0–3731.0)	636.0 (301.0–2160.0)	1238.0 (231.0–3332.0)	0.926
CK–MB, ng/ml	23.3 (3.0–112.4)	21.7 (2.0–58.6)	23.3 (3.4–146.7)	0.390
Myoglobin, ng/ml	315.1 (95.0–1200.0)	161.7 (94.5–301.8)	289.7 (91.8–1200.0)	0.372
Lactate dehydrogenase, U/L	424.0 (247.0–834.0)	323.0 (218.0–391.0)	487.5 (326.0–863.8)	0.070
hs CRP, mg/l	2.2 (0.5–3.9)	1.3 (0.6–2.8)	2.2 (0.4–3.1)	0.701
MDAAT cardiac activity VAS	1.3 ± 0.9	–	1.7 ± 0.6	–
Clinical cardiovascular symptoms	2 (4)	–	2 (5)	
Abnormal hs cTnI (M, > 34.2 pg/ml, F, > 15.6 pg/ml), n (%)	14 (29)	0 (0)	14 (38)	–
Abnormal electrocardiography, n (%)	24 (49)	0 (0)	24 (65)	–
Abnormal standard echocardiography, n (%)	9 (18)	0 (0)	9 (24)	–
Abnormal standard CMR, n (%)	30 (61)	0 (0)	30 (81)	–

Data are expressed as N (%), mean ± standard deviation, or median (interquartile range).

IIM, idiopathic inflammatory myopathy; PM, polymyositis; DM, dermatomyositis; IMNM, immune-mediated necrotizing myopathy; BMI, body mass index; SD, standard deviation; IQR, interquartile; hs cTnI, high-sensitive cardiac troponin I; NT-proBNP, N-terminal pro brain natriuretic peptide; CK, creatine kinase; hs CRP, high-sensitive C reactive protein; NSAID, nonsteroidal anti-inflammatory drug; MDAAT, the Myositis Disease Activity Assessment Tool; VAS, visual analogue; CMR, cardiac magnetic resonance.

Bold value denote statistical significance (*p* < 0.05).

### Function, LGE, and strain by CMR

Compared to the healthy control group, all LV and RV volumetric and functional parameters showed no significant difference in IIM-C+ and IIM-C− groups (Table 2). All LGE lesions were detected in inferior-lateral and inferior wall, which were all distributed in subepicardium. No significant difference was found in LV global circumferential strain (GCS) (*p* = 0.097) among the three groups. Compared to the controls, LV global longitudinal strain (GLS) (adjusted *p* = 0.001), RV GCS and RV GLS of IIM-C^+^ patients were reduced significantly (adjusted *p* *˂* 0.001, and adjusted *p* *˂* 0.001). However, LV GLS, RV GCS and RV GLS did not show significant difference between the IIM-C^-^ and IIM-C^+^ groups (adjusted *p* *˃* 0.05 for all).

**Table 2:** CMR volume and functional parameters of controls, IIM patients without and with myocardial involvement.

Parameters	Controls (n = 25)	IIM-C^-^ (n = 12)	IIM-C^+^ (n = 37)	*p* values
LVEDVI, mL/m^2^	69.9 (61.4–83.0)	65.4 (60.2–74.5)	74.0 (68.1–81.0)	0.191
LVESVI, mL/m^2^	28.0 (21.7–33.1)	26.4 (22.4–29.5)	27.2 (21.3–34.5)	0.817
LVSVI, mL/m^2^	44.9 ± 8.1	41.1 ± 8.2	44.9 ± 8.6	0.363
LVEF, %	62.8 ± 6.5	61.0 ± 7.9	60.8 ± 10.7	0.561
LVmassI, g/m^2^	44.9 ± 10.2	39.7 ± 7.6	42.8 ± 9.8	0.389
RVEDVI, mL/m^2^	57.4 ± 13.6	58.3 ± 14.1	54.9 ± 11.9	0.629
RVESVI, mL/m^2^	24.8 (19.3–27.9)	24.4 (15.9–30.7)	20.6 (17.6–27.2)	0.846
RVSVI, mL/m^2^	33.4 ± 9.0	34.3 ± 9.3	31.8 ± 8.9	0.621
RVEF, %	58.4 ± 8.5	59.0 ± 9.3	57.7 ± 10.1	0.889
RVmassI, g/m^2^	14.5 ± 3.0	13.1 ± 3.0	14.7 ± 3.1	0.307
**CMR strain**				
LVGCS, %	− 23.6 ± 5.3	− 23.0 ± 7.2	− 20.7 ± 4.8	0.097
LVGLS, %	− 16.7 (− 22.1 to − 14.7)	− 14.3 (− 19.7 to − 9.2)	− 12.8 (− 15.3 to − 10.1)^#^	**0.001**
RVGCS, %	− 15.1 ± 2.7	− 11.5 ± 8.0	− 10.2 ± 5.2^#^	**0.002**
RVGLS, %	− 14.1 (− 17.3 to − 12.5)	− 10.6 (− 12.9 to − 8.8)	− 9.2 (− 12.0 to − 6.8)^#^	**˂** **0.001**

Data are expressed mean ± standard deviation, or median (interquartile range).

Bold value denotes *p* < 0.05 for ANOVA or Kruskal–Wallis test.

IIM, idiopathic inflammatory myopathy; CMR, cardiac magnetic resonance; LV, left ventricle; EDVI, end-diastolic volume index; ESVI, end-systolic volume index; SVI, stroke volume index; EF, ejection fraction; massI, mass index; RV, right ventricle; GCS, global circumferential strain; GLS, global longitudinal strain.

^#^Indicates adjusted *p* < 0.05 for controls versus IIM-C^+^.

^§^Indicates adjusted *p* < 0.05 for controls versus IIM-C^−^.

^&^Indicates adjusted *p* < 0.05 for IIM-C^−^ versus IIM-C^+^.

### Parameters of CMR T1 and T2 mapping

**Figure 3:**
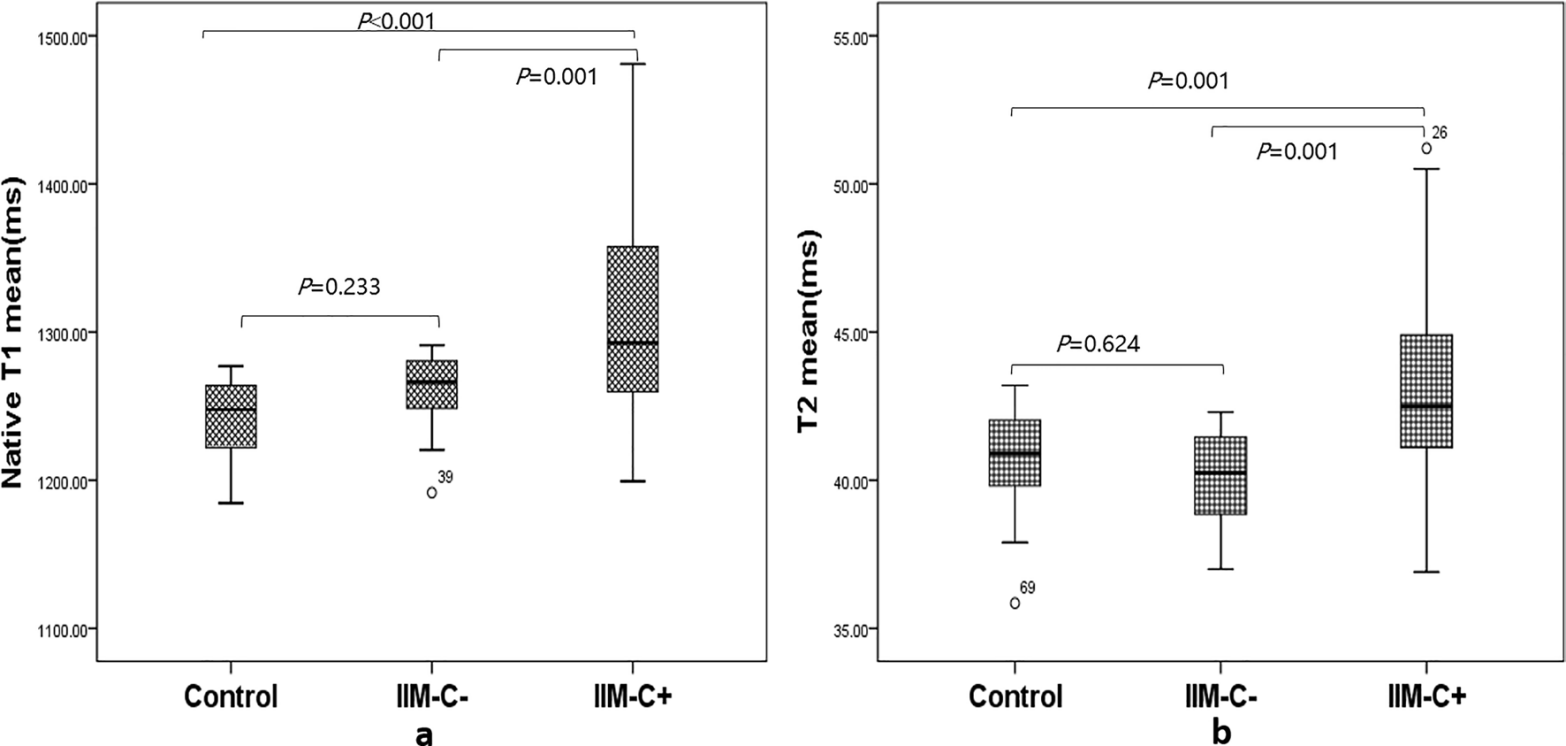
Boxplots showing the values of native T1 mean (**a**) and T2 mean (**b**) in the three classes of subjects.

Mean of native T1/T2 mapping and extracellular volume (ECV) are reported in Table 3. The native T1 and T2 mean showed higher values in IIM-C^+^ than in IIM-C^-^ (adjusted *p* ˂ 0.001, and adjusted *p* = 0.001, Fig. 3). Compared to the healthy controls, native T1 and T2 mean, as well as ECV, were all elevated significantly in IIM-C^+^ (adjusted *p* < 0.05 for all).

**Table 3:** CMR tissue characteristic parameters of controls, IIM patients without and with myocardial involvement.

Parameters	Controls (n = 25)	IIM-C− (n = 12)	IIM-C+ (n = 37)	*p* values
Native T1 mean, ms	1241.0 ± 26.0	1259.1 ± 29.4	1314.8 ± 68.9^#/&^	**< 0.001**
T2 mean, ms	40.7 ± 1.6	40.0 ± 1.7	43.1 ± 3.5^#/&^	**˂ 0.001**
ECV, %	24.5 ± 2.5	26.7 ± 4.0	29.2 ± 5.8^#^	**0.010**

Data are expressed mean ± standard deviation, or median (interquartile range).

Bold value denotes *P* < 0.05 for ANOVA or Kruskal–Wallis test.

IIM, idiopathic inflammatory myopathy; CMR, cardiac magnetic resonance; ECV, extracellular volume.

^#^Indicates adjusted *P* < 0.05 for controls versus IIM-C+.

^§^Indicates adjusted *P* < 0.05 for controls versus IIM-C−.

^&^Indicates adjusted *P* < 0.05 for IIM-C- versus IIM-C+.

**Figure 4:**
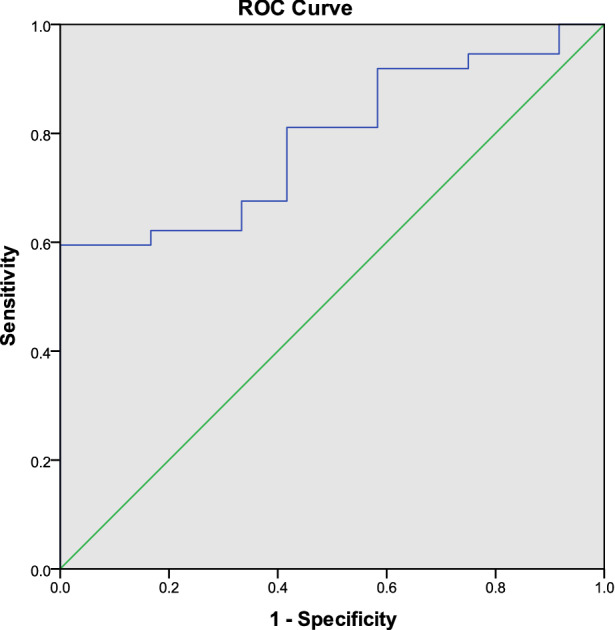
ROC analysis of combined T1 mean and T2 mean to screen cardiac involvement from IIM patients (AUC = 0.788, *p* = 0.003).

No significant differences were observed in native T1/T2 mean, and ECV between controls and IIM-C^-^ group (adjusted *p* > 0.05 for all). Combined T1 mean and T2 mean, the AUC of ROC curve was 0.788 (95% CI 0.660–0.916, *p* = 0.003) with sensitivity of 81.1% (Fig. 4).

### Multi-parametric CMR score for screening cardiac involvement in IIM

**Figure 5:**
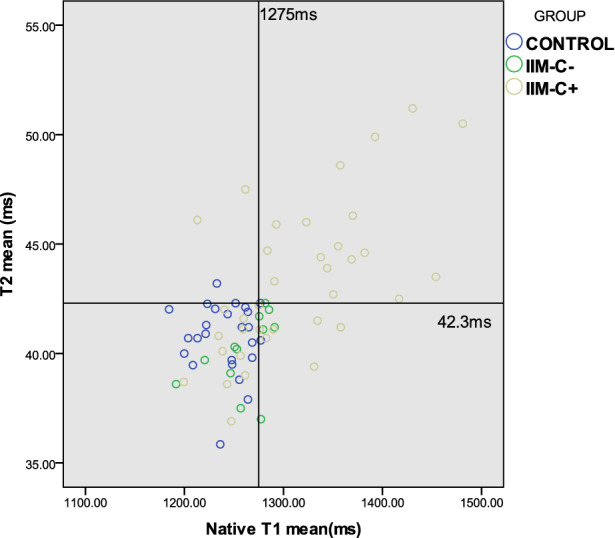
Scatter plots of T1 mean and T2 mean of control and IIM patients. The reference line of native T1 mean is 1275 ms, and the reference line of T2 mean is 42.3 ms.

For T1 mapping in healthy volunteers, the native T1 mean was 1241.0 ms, and the 95% upper quantile was 1274.9 ms. For T2 mapping in healthy volunteers, the T2 mean was 40.7 ms, and the 95% upper quantile was 42.3 ms. From this, pathologic thresholds for cardiac involvement in IIM in this study were determined as native T1 mean ≥ 1275 ms and /or T2 mean ≥ 42.3 ms (Fig. 5). Thirty-three of 49 (67%) IIM patients had a CMR score ≥ 1 (Table 4). McNemar’s test revealed no significant difference between CMR score and MDAAT (*p* = 0.454, Table 4).

**Table 4:** Contingency table and McNemar test of CMR score and MDAAT.

Count	MDAAT No cardiac involvement	MDAAT Cardiac involvement	Total
CMR score No cardiac involvement	6	10	16
CMR score Cardiac involvement	6	27	33
Total	12	37	49

## Discussion

The major finding of our study is that a non-invasive, contrast-free, multi-parametric quantitative CMR score could screen for cardiac involvement in IIM patients, compared to MDAAT. The CMR score may help identify IIM patients with cardiac involvement at an early stage, and facilitate timely treatment to prevent further adverse myocardial remodelling. As our CMR score only requires native T1 and T2-mapping, it can also be applied to the IIM patients with contraindications for GBCA.

Routine functional parameters of the left ventricle are known to be insensitive to subtle cardiac abnormalities in patients with preserved LVEF^14^. In recent years, T1 and T2 mapping emerged as important techniques to assess myocardial tissue characteristics in terms of tissue fibrosis and inflammation. In this study, while the goal is to diagnose myocardial involvement from IIM patients, we included an additional cohort of healthy controls as a reference to study the changes in myocardial tissue characteristics caused by the IIM disease.

In this study myocardial native T1 mean and ECV were all elevated significantly in IIM patients with myocardial involvement compared to healthy controls, suggesting that diffuse myocardial fibrosis has developed in IIM. Myocardial T2 mean was found elevated in the IIM-C+ group compared to the IIM-C- group, and T2 mean of the IIM-C+ group was also above the 95% quantile in the healthy control cohort, which suggests myocardial edema in case of myocardial involvement^24^.

Another interesting finding of this study is the RV involvement in the IIM cohort as manifested by reduced RV strain compared to the healthy controls. Strain parameters have been found to be more sensitive than EF to reflect early ventricular dysfunction^25^. However, studies investigating RV myocardial deformation in IIM are rare. In our cohort, RV involvement may not be caused by intrinsic RV disorders, but by systemic inflammation and endothelial dysfunction of pulmonary vessels as secondary consequences of IIM^25^. Compared to IIM-C^-^ group, nevertheless, all biventricular strains in IIM-C^+^ did not exhibit significant differences.

There are several limitations in the present study. First of all, the study lacked a golden standard, i.e. endomyocardial biopsy, to quantify myocardial involvement in IIM. Instead, we used the commonly used clinical criteria including MDAAT, cardiac-specific serological markers, electrocardiography, and echocardiography ^8,26^. Another limitation is the small sample size in the IIM-C+ and IIM-C− groups due to the low prevalence of the disease. Furthermore, this is a single center study, in which the native T1/T2 values can be specific to the MRI device. Nevertheless, we showed that in principle, a combination of quantitative evaluation of diffused fibrosis, and inflammation in the myocardium can be a sensitive tool to identify subclinical cardiac involvement in IIM cohort. Future studies are warranted to further validate this CMR score with more extensive patient data.

In conclusion, we have developed a multi-parametric CMR score (native T1 mean and T2 mean), which may screen for cardiac involvement in IIM patients, compared to MDAAT. The CMR score only requires native T1 and T2 parameters, without the need for contrast agent administration and is therefore applicable in patients with contraindications for GBCA. Further study should be performed to validate our CMR score accuracy, which potentially facilitates early treatment to prevent cardiac mortality in IIM patient group.
